# 2-*tert*-Butyl-4-chloro-5-[4-(2-fluoro­eth­oxy)benz­yloxy]pyridazin-3(2*H*)-one

**DOI:** 10.1107/S1600536812020491

**Published:** 2012-05-12

**Authors:** Huihui Jing, Tiantian Mou, Xianzhong Zhang

**Affiliations:** a Key Laboratory of Radiopharmaceuticals, Ministry of Education, College of Chemistry, Beijing Normal University, 19 Xinjiekou Outer St, Beijing 100875, People’s Republic of China

## Abstract

In the title compound, C_17_H_20_ClFN_2_O_3_, the dihedral angle between the pyridazine and benzene rings is 41.37 (10)°. In the crystal, there are no significant intermolecular interactions present. The terminal –CH_2_F group is disordered over two sets of sites with an occupancy ratio of 0.737 (2):0.263 (2).

## Related literature

For details of the synthesis, see: Mou *et al.* (2010 ▶, 2012 ▶). For possible applications of the title compound as a myocardial perfusion imaging agent for positron emission tomography (when labelled with ^18^F), see: Mou *et al.* (2011 ▶); Mou *et al.* (2012 ▶).
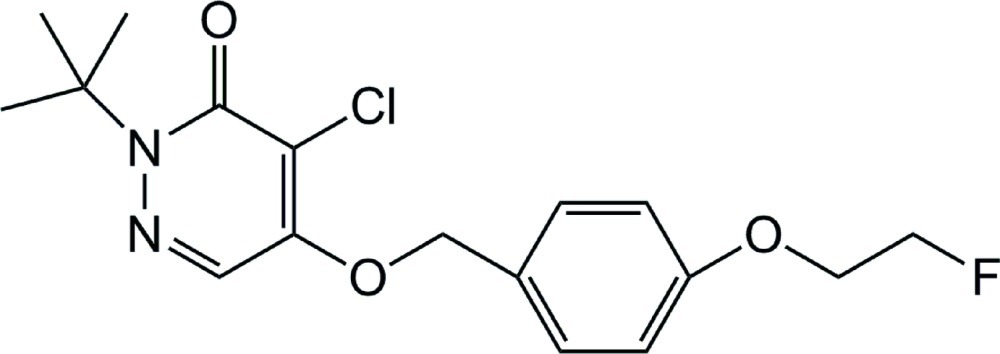


## Experimental

### 

#### Crystal data

C_17_H_20_ClFN_2_O_3_
*M* *_r_* = 354.80
Triclinic, 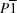

*a* = 8.7170 (14) Å
*b* = 9.5850 (16) Å
*c* = 11.8524 (19) Å
α = 110.475 (2)°
β = 107.185 (2)°
γ = 96.424 (3)°
*V* = 860.3 (2) Å^3^
*Z* = 2
Mo *K*α radiation
μ = 0.25 mm^−1^
*T* = 150 K
0.47 × 0.38 × 0.35 mm

#### Data collection

Bruker APEXII CCD area-detector diffractometer
Absorption correction: multi-scan (*SADABS*; Bruker, 2009 ▶) *T*_min_ = 0.892, *T*_max_ = 0.918
4337 measured reflections
3090 independent reflections
2788 reflections with *I* > 2σ(*I*)
*R*_int_ = 0.016

#### Refinement

*R*[*F* ^2^ > 2σ(*F* ^2^)] = 0.036
*wR*(*F* ^2^) = 0.094
*S* = 1.05
3090 reflections
227 parameters
H-atom parameters constrained
Δρ_max_ = 0.32 e Å^−3^
Δρ_min_ = −0.28 e Å^−3^

### 

Data collection: *APEX2* (Bruker, 2009 ▶); cell refinement: *SAINT* (Bruker, 2009 ▶); data reduction: *SAINT*; program(s) used to solve structure: *SHELXS97* (Sheldrick, 2008 ▶); program(s) used to refine structure: *SHELXL97* (Sheldrick, 2008 ▶); molecular graphics: *SHELXTL* (Sheldrick, 2008 ▶); software used to prepare material for publication: *SHELXTL*.

## Supplementary Material

Crystal structure: contains datablock(s) I, global. DOI: 10.1107/S1600536812020491/aa2046sup1.cif

Structure factors: contains datablock(s) I. DOI: 10.1107/S1600536812020491/aa2046Isup4.hkl

Supplementary material file. DOI: 10.1107/S1600536812020491/aa2046Isup3.cml

Additional supplementary materials:  crystallographic information; 3D view; checkCIF report

## Figures and Tables

**Fig. 1.**
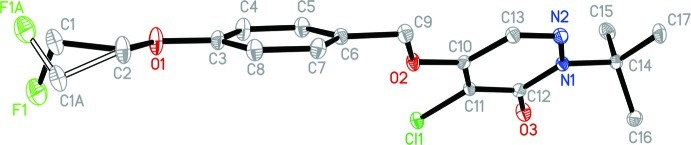
Molecular structure of the title compound showing the atom labeling scheme and 30% probability displacement ellipsoids. H atoms are omitted for clarity.

**Fig. 2.**
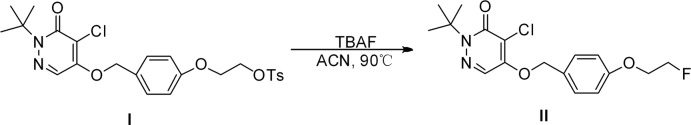
The synthesis route of the title compound.

## supplementary crystallographic information

### Comment

Myocardial uptake of the pyridaben analogues, which is correlated with blood flow, makes them potential myocardial perfusion imaging (MPI) agents for the positron emission tomography (PET) when labeled with ^18^F (Mou *et al.*, 2010; Mou *et al.*, 2011; Mou *et al.*, 2012). Thus, the development of pyridaben analogues may lead to discover new valuable PET MPI agents.

The molecular structure of the title compound (measured at 150 K) is shown in Fig. 1. The dihedral angle between the pyridazine ring and the benzene ring is 41.37 (10)°. The terminal CH_2_F group is disordered between two positions with occupancies 0.737 (2) for C1F1 and 0.263 (2) for C1AF1A.

### Experimental

The synthesis route is shown in Fig. 2. The solution of *tert*-butylammonium fluoride (1 mmol in 1 ml tetrahydrofuran) was stirred in a stream of nitrogen at 110 °C to remove the solvent. Then 2-*tert*-butyl-4-chloro-5-(4-(2-tosylethoxy-ethoxy)-benzyloxy)-2*H*-pyridazin-3-one (compound I, 0.30 mmol in 3 ml anhydrous CH_3_CN) was added to the above evaporation residue, and refluxed for 40 min at 90 °C. After concentration under reduced pressure, the residue was chromatographed over a column of silica gel and eluted with the mixture of dichloromethane and methanol (100:1). The product was obtained as white solid. The product was then recrystallized from the mixture of hexane and methanol (2:1) yielding colorless crystals of the title compound suitable for the single-crystal X-ray diffraction.

### Refinement

The H atoms bound to C atoms were positioned geometrically and refined using a riding model, with C—H = 0.99 Å for CH_2_ groups, 0.95 Å for aryl and 0.98 Å for methyl H atoms, *U*_iso_(H) =1.2*U*_eq_(C) for CH_2_ groups and aryl, and *U*_iso_(H) =1.5*U*_eq_(C) for methyl H atoms.

### Crystal data

**Table:**

C_17_H_20_ClFN_2_O_3_	*Z* = 2
*M**_r_* = 354.80	*F*(000) = 372
Triclinic, *P*1	*D*_x_ = 1.370 Mg m^−^^3^
Hall symbol: -P 1	Melting point: 396 K
*a* = 8.7170 (14) Å	Mo *K*α radiation, λ = 0.71073 Å
*b* = 9.5850 (16) Å	Cell parameters from 2518 reflections
*c* = 11.8524 (19) Å	θ = 2.3–27.5°
α = 110.475 (2)°	µ = 0.25 mm^−^^1^
β = 107.185 (2)°	*T* = 150 K
γ = 96.424 (3)°	Column, colourless, colourless
*V* = 860.3 (2) Å^3^	0.47 × 0.38 × 0.35 mm

### Data collection

**Table:**

Bruker APEXII CCD area-detector diffractometer	3090 independent reflections
Radiation source: fine-focus sealed tube	2788 reflections with *I* > 2σ(*I*)
Graphite monochromator	*R*_int_ = 0.016
phi and ω scans	θ_max_ = 25.3°, θ_min_ = 2.3°
Absorption correction: multi-scan (*SADABS*; Bruker, 2009)	*h* = −6→10
*T*_min_ = 0.892, *T*_max_ = 0.918	*k* = −11→10
4337 measured reflections	*l* = −14→13

### Refinement

**Table:**

Refinement on *F*^2^	Primary atom site location: structure-invariant direct methods
Least-squares matrix: full	Secondary atom site location: difference Fourier map
*R*[*F*^2^ > 2σ(*F*^2^)] = 0.036	Hydrogen site location: inferred from neighbouring sites
*wR*(*F*^2^) = 0.094	H-atom parameters constrained
*S* = 1.05	*w* = 1/[σ^2^(*F*_o_^2^) + (0.0431*P*)^2^ + 0.4066*P*] where *P* = (*F*_o_^2^ + 2*F*_c_^2^)/3
3090 reflections	(Δ/σ)_max_ < 0.001
227 parameters	Δρ_max_ = 0.32 e Å^−^^3^
0 restraints	Δρ_min_ = −0.28 e Å^−^^3^

### Special details

**Table:**

Geometry. All s.u.'s (except the s.u. in the dihedral angle between two l.s. planes) are estimated using the full covariance matrix. The cell s.u.'s are taken into account individually in the estimation of s.u.'s in distances, angles and torsion angles; correlations between s.u.'s in cell parameters are only used when they are defined by crystal symmetry. An approximate (isotropic) treatment of cell s.u.'s is used for estimating s.u.'s involving l.s. planes.
Refinement. Refinement of *F*^2^ against ALL reflections. The weighted *R*-factor *wR* and goodness of fit *S* are based on *F*^2^, conventional *R*-factors *R* are based on *F*, with *F* set to zero for negative *F*^2^. The threshold expression of *F*^2^ > σ(*F*^2^) is used only for calculating *R*-factors(gt) *etc*. and is not relevant to the choice of reflections for refinement. *R*-factors based on *F*^2^ are statistically about twice as large as those based on *F*, and *R*- factors based on ALL data will be even larger.

### Fractional atomic coordinates and isotropic or equivalent isotropic displacement parameters (Å^2^)

**Table:**

	*x*	*y*	*z*	*U*_iso_*/*U*_eq_	Occ. (<1)
C1	0.7610 (4)	1.6895 (3)	0.6672 (3)	0.0356 (7)	0.737 (2)
H1A	0.7483	1.7463	0.7502	0.043*	0.737 (2)
H1B	0.8756	1.7279	0.6751	0.043*	0.737 (2)
F1	0.6489 (2)	1.71415 (19)	0.56807 (19)	0.0563 (5)	0.737 (2)
C1A	0.6652 (12)	1.6621 (10)	0.6292 (9)	0.0356 (7)	0.263 (2)
H1A1	0.5919	1.6389	0.5400	0.043*	0.263 (2)
H1A2	0.6036	1.6957	0.6880	0.043*	0.263 (2)
F1A	0.8073 (7)	1.7730 (6)	0.6664 (5)	0.0563 (5)	0.263 (2)
C2	0.7295 (3)	1.5215 (2)	0.63796 (19)	0.0357 (5)	
H2A	0.7927	1.5042	0.7142	0.043*	
H2B	0.6104	1.4780	0.6141	0.043*	
C3	0.7804 (2)	1.29834 (18)	0.49589 (16)	0.0253 (4)	
C4	0.8406 (2)	1.2381 (2)	0.39752 (17)	0.0301 (4)	
H4	0.8787	1.3019	0.3607	0.036*	
C5	0.8452 (2)	1.08544 (19)	0.35314 (16)	0.0278 (4)	
H5	0.8883	1.0456	0.2867	0.033*	
C6	0.7879 (2)	0.98912 (18)	0.40407 (15)	0.0224 (4)	
C7	0.7268 (2)	1.05060 (19)	0.50116 (16)	0.0267 (4)	
H7	0.6861	0.9860	0.5363	0.032*	
C8	0.7234 (2)	1.2049 (2)	0.54876 (17)	0.0276 (4)	
H8	0.6827	1.2454	0.6165	0.033*	
C9	0.7940 (2)	0.82347 (19)	0.35594 (16)	0.0271 (4)	
H9A	0.9074	0.8134	0.3944	0.032*	
H9B	0.7176	0.7643	0.3796	0.032*	
C10	0.74817 (19)	0.62230 (17)	0.15061 (16)	0.0205 (3)	
C11	0.69499 (19)	0.56701 (17)	0.01958 (15)	0.0196 (3)	
C12	0.69623 (19)	0.41371 (18)	−0.05951 (15)	0.0205 (3)	
C13	0.8067 (2)	0.52043 (18)	0.20730 (16)	0.0244 (4)	
H13	0.8450	0.5560	0.2986	0.029*	
C14	0.7659 (2)	0.16475 (18)	−0.05844 (16)	0.0224 (4)	
C15	0.8931 (2)	0.1704 (2)	−0.12319 (19)	0.0319 (4)	
H15A	0.8604	0.2209	−0.1825	0.048*	
H15B	0.8984	0.0657	−0.1714	0.048*	
H15C	1.0020	0.2281	−0.0571	0.048*	
C16	0.5943 (2)	0.06939 (19)	−0.15633 (18)	0.0324 (4)	
H16A	0.5141	0.0780	−0.1127	0.049*	
H16B	0.5978	−0.0383	−0.1945	0.049*	
H16C	0.5612	0.1075	−0.2244	0.049*	
C17	0.8217 (2)	0.09524 (19)	0.03935 (18)	0.0308 (4)	
H17A	0.9307	0.1564	0.1034	0.046*	
H17B	0.8286	−0.0101	−0.0049	0.046*	
H17C	0.7419	0.0946	0.0827	0.046*	
Cl1	0.62207 (5)	0.67867 (4)	−0.06075 (4)	0.02475 (13)	
N1	0.75585 (16)	0.32789 (14)	0.01074 (12)	0.0199 (3)	
N2	0.81035 (17)	0.38114 (15)	0.14042 (13)	0.0241 (3)	
O1	0.78194 (18)	1.45135 (14)	0.53292 (12)	0.0356 (3)	
O2	0.74477 (15)	0.76678 (12)	0.21681 (10)	0.0246 (3)	
O3	0.64984 (16)	0.36158 (13)	−0.17774 (11)	0.0292 (3)	

### Atomic displacement parameters (Å^2^)

**Table:**

	*U*^11^	*U*^22^	*U*^33^	*U*^12^	*U*^13^	*U*^23^
C1	0.0526 (19)	0.0305 (15)	0.0392 (17)	0.0225 (17)	0.0274 (16)	0.0189 (13)
F1	0.0628 (11)	0.0390 (9)	0.0698 (12)	0.0205 (8)	0.0142 (9)	0.0304 (9)
C1A	0.0526 (19)	0.0305 (15)	0.0392 (17)	0.0225 (17)	0.0274 (16)	0.0189 (13)
F1A	0.0628 (11)	0.0390 (9)	0.0698 (12)	0.0205 (8)	0.0142 (9)	0.0304 (9)
C2	0.0596 (13)	0.0248 (9)	0.0333 (10)	0.0188 (9)	0.0280 (10)	0.0119 (8)
C3	0.0340 (10)	0.0181 (8)	0.0221 (8)	0.0079 (7)	0.0098 (7)	0.0057 (7)
C4	0.0469 (11)	0.0229 (9)	0.0271 (9)	0.0100 (8)	0.0203 (8)	0.0114 (7)
C5	0.0380 (10)	0.0247 (9)	0.0220 (9)	0.0098 (7)	0.0151 (8)	0.0068 (7)
C6	0.0266 (9)	0.0197 (8)	0.0159 (8)	0.0058 (7)	0.0038 (7)	0.0048 (6)
C7	0.0355 (10)	0.0225 (8)	0.0239 (9)	0.0055 (7)	0.0131 (7)	0.0097 (7)
C8	0.0378 (10)	0.0253 (9)	0.0225 (9)	0.0104 (7)	0.0160 (8)	0.0075 (7)
C9	0.0376 (10)	0.0228 (9)	0.0176 (8)	0.0094 (7)	0.0076 (7)	0.0059 (7)
C10	0.0196 (8)	0.0163 (7)	0.0229 (8)	0.0034 (6)	0.0071 (7)	0.0057 (7)
C11	0.0197 (8)	0.0184 (8)	0.0220 (8)	0.0052 (6)	0.0076 (6)	0.0095 (7)
C12	0.0212 (8)	0.0206 (8)	0.0207 (8)	0.0056 (6)	0.0095 (7)	0.0077 (7)
C13	0.0294 (9)	0.0203 (8)	0.0178 (8)	0.0068 (7)	0.0044 (7)	0.0042 (7)
C14	0.0243 (8)	0.0166 (8)	0.0237 (9)	0.0072 (6)	0.0085 (7)	0.0046 (7)
C15	0.0329 (10)	0.0327 (10)	0.0375 (10)	0.0167 (8)	0.0188 (8)	0.0147 (8)
C16	0.0280 (10)	0.0188 (8)	0.0366 (10)	0.0057 (7)	0.0058 (8)	0.0003 (8)
C17	0.0422 (11)	0.0195 (8)	0.0308 (10)	0.0119 (8)	0.0126 (8)	0.0094 (7)
Cl1	0.0323 (2)	0.0221 (2)	0.0243 (2)	0.01014 (16)	0.01102 (17)	0.01263 (17)
N1	0.0233 (7)	0.0167 (7)	0.0173 (7)	0.0053 (5)	0.0062 (6)	0.0048 (5)
N2	0.0277 (8)	0.0217 (7)	0.0188 (7)	0.0063 (6)	0.0046 (6)	0.0065 (6)
O1	0.0646 (9)	0.0198 (6)	0.0329 (7)	0.0160 (6)	0.0302 (7)	0.0104 (5)
O2	0.0353 (7)	0.0166 (6)	0.0177 (6)	0.0091 (5)	0.0069 (5)	0.0035 (5)
O3	0.0432 (7)	0.0261 (6)	0.0187 (6)	0.0131 (5)	0.0117 (5)	0.0075 (5)

### Geometric parameters (Å, º)

**Table:**

C1—F1	1.400 (4)	C9—H9B	0.9900
C1—C2	1.497 (3)	C10—O2	1.3405 (19)
C1—H1A	0.9900	C10—C11	1.361 (2)
C1—H1B	0.9900	C10—C13	1.426 (2)
C1A—F1A	1.383 (11)	C11—C12	1.444 (2)
C1A—C2	1.541 (9)	C11—Cl1	1.7215 (16)
C1A—H1A1	0.9900	C12—O3	1.2276 (19)
C1A—H1A2	0.9900	C12—N1	1.400 (2)
C2—O1	1.425 (2)	C13—N2	1.302 (2)
C2—H2A	0.9900	C13—H13	0.9500
C2—H2B	0.9900	C14—N1	1.5169 (19)
C3—O1	1.373 (2)	C14—C17	1.518 (2)
C3—C8	1.385 (2)	C14—C15	1.529 (2)
C3—C4	1.388 (2)	C14—C16	1.531 (2)
C4—C5	1.381 (2)	C15—H15A	0.9800
C4—H4	0.9500	C15—H15B	0.9800
C5—C6	1.390 (2)	C15—H15C	0.9800
C5—H5	0.9500	C16—H16A	0.9800
C6—C7	1.383 (2)	C16—H16B	0.9800
C6—C9	1.501 (2)	C16—H16C	0.9800
C7—C8	1.393 (2)	C17—H17A	0.9800
C7—H7	0.9500	C17—H17B	0.9800
C8—H8	0.9500	C17—H17C	0.9800
C9—O2	1.450 (2)	N1—N2	1.3469 (18)
C9—H9A	0.9900		
			
F1—C1—C2	109.6 (2)	H9A—C9—H9B	108.6
F1—C1—H1A	109.8	O2—C10—C11	118.74 (14)
C2—C1—H1A	109.8	O2—C10—C13	124.85 (14)
F1—C1—H1B	109.8	C11—C10—C13	116.40 (14)
C2—C1—H1B	109.8	C10—C11—C12	122.61 (14)
H1A—C1—H1B	108.2	C10—C11—Cl1	121.01 (12)
F1A—C1A—C2	103.9 (6)	C12—C11—Cl1	116.38 (12)
F1A—C1A—H1A1	111.0	O3—C12—N1	122.25 (14)
C2—C1A—H1A1	111.0	O3—C12—C11	123.81 (15)
F1A—C1A—H1A2	111.0	N1—C12—C11	113.94 (14)
C2—C1A—H1A2	111.0	N2—C13—C10	123.44 (15)
H1A1—C1A—H1A2	109.0	N2—C13—H13	118.3
O1—C2—C1	106.72 (17)	C10—C13—H13	118.3
O1—C2—C1A	110.7 (4)	N1—C14—C17	109.19 (13)
O1—C2—H2A	110.4	N1—C14—C15	108.07 (13)
C1—C2—H2A	110.4	C17—C14—C15	109.53 (14)
C1A—C2—H2A	130.3	N1—C14—C16	109.30 (13)
O1—C2—H2B	110.4	C17—C14—C16	108.73 (14)
C1—C2—H2B	110.4	C15—C14—C16	111.99 (15)
C1A—C2—H2B	81.7	C14—C15—H15A	109.5
H2A—C2—H2B	108.6	C14—C15—H15B	109.5
O1—C3—C8	124.76 (15)	H15A—C15—H15B	109.5
O1—C3—C4	115.27 (15)	C14—C15—H15C	109.5
C8—C3—C4	119.97 (15)	H15A—C15—H15C	109.5
C5—C4—C3	120.07 (16)	H15B—C15—H15C	109.5
C5—C4—H4	120.0	C14—C16—H16A	109.5
C3—C4—H4	120.0	C14—C16—H16B	109.5
C4—C5—C6	121.06 (16)	H16A—C16—H16B	109.5
C4—C5—H5	119.5	C14—C16—H16C	109.5
C6—C5—H5	119.5	H16A—C16—H16C	109.5
C7—C6—C5	118.15 (15)	H16B—C16—H16C	109.5
C7—C6—C9	121.02 (15)	C14—C17—H17A	109.5
C5—C6—C9	120.83 (15)	C14—C17—H17B	109.5
C6—C7—C8	121.73 (16)	H17A—C17—H17B	109.5
C6—C7—H7	119.1	C14—C17—H17C	109.5
C8—C7—H7	119.1	H17A—C17—H17C	109.5
C3—C8—C7	119.02 (16)	H17B—C17—H17C	109.5
C3—C8—H8	120.5	N2—N1—C12	124.27 (13)
C7—C8—H8	120.5	N2—N1—C14	115.40 (12)
O2—C9—C6	107.02 (13)	C12—N1—C14	120.33 (13)
O2—C9—H9A	110.3	C13—N2—N1	119.34 (14)
C6—C9—H9A	110.3	C3—O1—C2	117.83 (13)
O2—C9—H9B	110.3	C10—O2—C9	118.79 (12)
C6—C9—H9B	110.3		
